# 

**Published:** 1881-04-20

**Authors:** 


Entered at the Post-Office at Atlanta as second-class matter.

VOL. XI. APRIL 20, 1881. NO. 4.

THE

### SOUTHERN MEDICAL RECORD:

### A MONTHLY JOURNAL OF PRACTICAL MEDICINE.

Terms: $2 00 per Annum, in Advance; 4 Copies $6.00; Single Copies 20 Cents.

*"QUICQUID PRÆCIPIES ESTO BREVIS."*

Address R. C. WORD, M.D., Managing Editor, Atlanta, Ga.

